# Trends of the second measles vaccine (MCV2) over time after its launch as part of routine immunization in Nigeria: a brief research report

**DOI:** 10.3389/fpubh.2024.1392996

**Published:** 2024-10-28

**Authors:** Ryoko Sato

**Affiliations:** Independent Researcher, Boston, MA, United States

**Keywords:** measles, measles containing vaccine, MCV, routine immunization, Nigeria

## Abstract

**Introduction:**

Nigeria has one of the highest measles burdens and the lowest vaccination coverage in the world. Geographical disparity in the coverage has also been persistent. Between 2019 and 2021, the Nigerian government introduced the second Measles vaccine (MCV2) into routine immunization (RI). This study evaluated the trends of the MCV2 coverage over time across geographical zones.

**Methods:**

The monthly data on the MCV2 coverage from District Health Information Software (DHIS2) for all the health facilities in Nigeria were aggregated by the geopolitical zone, and the trend of the MCV2 coverage was analyzed over time.

**Results:**

The MCV2 coverage in each zone was approximately 20% when the MCV2 program was launched. The MCV2 coverage was higher in the northern zones (35–42%) than in the southern zones (22–31%) 1 year after the launch. Similarly, at 2.5 years, the MCV2 coverage ranged from 38 to 46% in northern zones, while in southern zones, it ranged from 23 to 37%.

**Discussion and conclusion:**

The introduction of MCV2 as part of the RI schedule potentially narrows down the health inequity in Nigeria.

## Introduction

Vaccination saves lives (1). Measles vaccination, in particular, is one of the most efficient interventions (2). Before the discovery of the measles vaccine in the United States in the 1960s, measles caused 3 million deaths annually (3). However, with the vaccine’s introduction, deaths dropped to under 100 cases in the United States (3).

Despite free access to the highly effective measles vaccine, Nigeria continues to experience one of the highest measles burdens and lowest vaccine uptake rates globally (4, 5).

There is also a significant disparity within Nigeria. The northern and southern zones differ socially, demographically, and economically. Southern zones are generally better off. For example, 60% of the poor live in the north, while the rest live in the south (6). The infant mortality rate in Nigeria is among the highest in the world, with northern zones showing a higher rate (62 per 1,000 live births) than southern zones (30 per 1,000 live births) (7). Vaccination coverage at the national level follows a similar trend: The overall full immunization rate among children aged 12–23 months is 31.3%, one of the lowest globally. In southern zones, the full immunization rate is 43–57%, whereas northern zones achieve only 20–31% (8).

Between 2019 and 2021, the Nigerian government introduced the second dose of the measles vaccine (MCV2) as part of the routine immunization (RI) campaign (9). Before this, the first dose (MCV1) had been part of the RI where children received MCV1 at the age of 9 months (9, 10).

Given the vaccination disparity between northern and southern Nigeria, studying vaccination trends in different geopolitical zones after the MCV2 introduction is crucial.

This study evaluated MCV2 coverage across geographical zones before and after its introduction into the RI schedule.

## Methods

### Introduction of second measles vaccine (MCV2)

**Figure 1:**
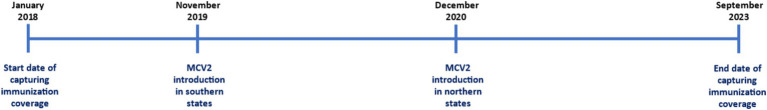
Timeline of MCV2 introduction.

MCV2 was first introduced in Nigeria’s southern zones in November 2019, followed by the northern zones between December 2020 and January 2021 (Figure 1). The Nigerian federal government introduced MCV2 as part of the RI schedule. Currently, it is administered to children aged 15–24 months, while MCV1, also part of the RI schedule, is given at 9 months.

Before MCV2 was part of the RI schedule, it was only available during the measles campaign. Children were eligible to receive MCV2 before its introduction if they were 24 months or older at the time of RI launch in each zone before MCV2 was part of the RI schedule.

### Data

Health-facility-level data, collected by the Health Management Information System through the District Health Information Software (DHIS2) platform, were used at the aggregated state level. DHIS2 provides monthly information on the quantity of various health services provided by public health facilities across Nigeria. This study uses secondary data collected via DHIS2 with access granted by the Nigeria Primary Healthcare Development Agency. The ethical approval process was waived as the data did not contain identifiable information.

The primary outcome for the analysis was the monthly NCV2 coverage from January 2018 to September 2023, aggregated by geopolitical zone. MCV2 coverage was calculated based on the number of MCV2 doses given divided by the eligible population based on age. However, since Nigeria’s last census was in 2006, the eligible population estimate is subject to error due to projections. The number of doses is reported by each RI facility and collated nationally. MCV2 doses were intended for children aged 15–24 months old, assuming no errors in age reporting. Nigeria’s 36 states and the Federal Capital Territory are grouped into six zones: north central, northeast, northwest, southeast, south–south, and southwest. Northern states comprise the first three zones, while the others are grouped as southern states.

**Figure A1:**
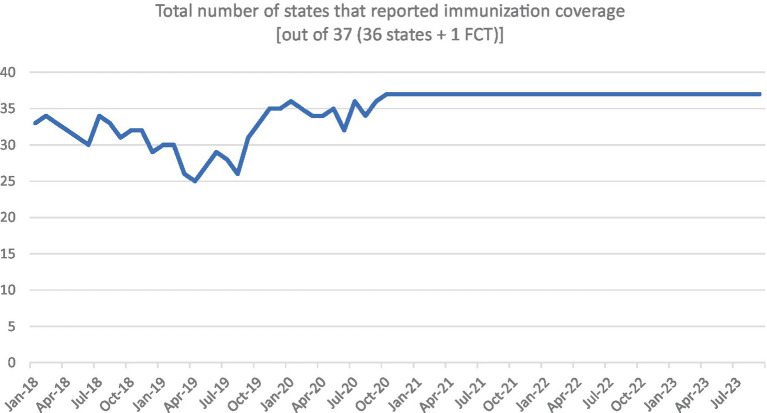
Number of states with reported immunization coverage.

Figure A1 shows the total number of states that reported immunization coverage over time.

### Analysis

This study provides a descriptive analysis of MCV2 coverage trends by zone from 2018 to 2023 (Figure 1). The overall trend of the MCV2 coverage was plotted over time by zone. The difference in the vaccine uptake was assessed using *t*-tests. Data analysis was performed using STATA version 18.

## Results

**Figure 2:**
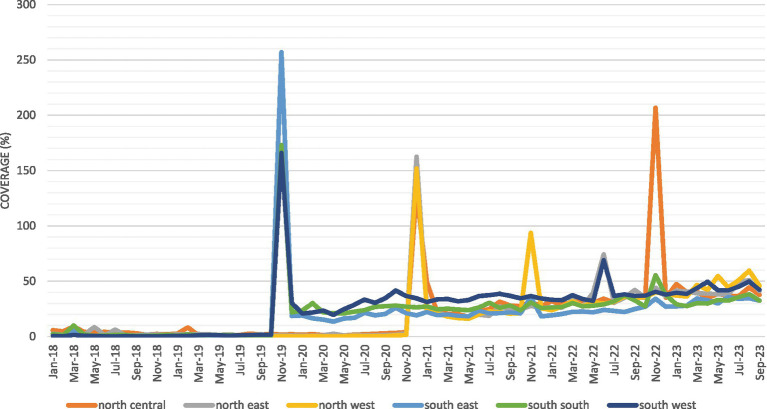
High-frequency MCV2 dose coverage over time by geopolitical zone (%).

Figure 2 presents the monthly pattern of the MCV2 coverage by geopolitical zone. There were several spikes in coverage: the first was driven by southern states in November 2019 and the second was driven by northern states in December 2020. These spikes exceeded 100%, suggesting that MCV2 was given to children, including some ineligible at the time. Overall, from 2018 to 2023, the figure shows an upward trend. Before the launch, MCV2 coverage was 1.3% in October 2019, rising to 39.0% by September 2023 (*p* < 0.001).

**Figure 3:**
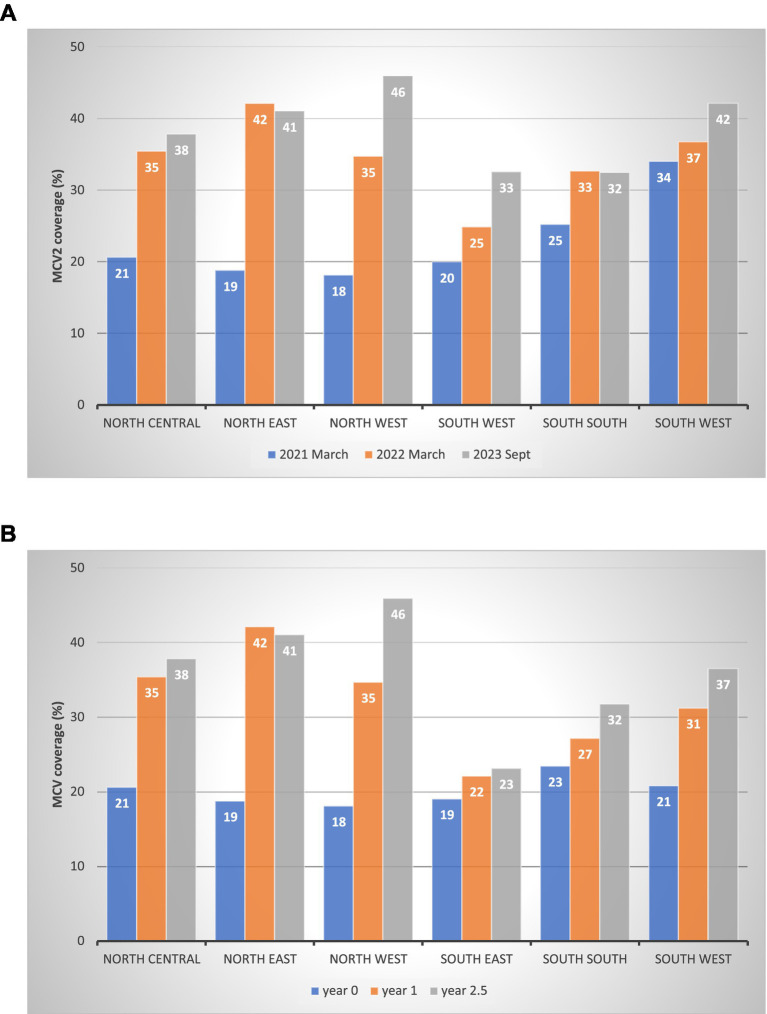
MCV2 coverage at three points in time by zone. (A) MCV2 coverage post-launch: absolute time by calendar year by geopolitical zone (%). (B) MCV2 coverage: relative time from the launch date by geopolitical zone (%).

Figure 3A presents the MCV2 coverage at three points (absolute time in calendar year): (a) right after the northern launch in March 2021 (1 year and 2 months after the southern launch in January 2020), (b) one year later in March 2022, and (c) the latest data point in September 2023. In March 2021, southern zones had higher coverage than northern zones: 34% in the southwest zone, 25% in the south–south zone, and 20% in the southwest zone. In northern zones, the highest was 21% in the north central zone, followed by 19% in the northeast and 18% in the northwest zones. By March 2022, northern zones had higher coverage: 42% in the northeast, 35% in the north central and northwest. The southern zone had 37% in the southwest, 33% in the south–south, and 25% in the southwest.

As explained in the Methods section, the MCV2 launch timing differed by zone, with the southern zone introducing MCV2 earlier than the northern. Figure 3B presents MCV2 coverage at year 0, year 1, and year 2.5 since each zone’s launch (relative time). At year 0, the coverage was similar across zones, approximately 20%. By year 1, the coverage was higher in the northern zones (35–42%) than in the southern zones (22–31%). At year 2.5, the pattern persisted with increased coverage: 38–46% in northern zones and 23–37% in southern zones.

**Figure 4:**
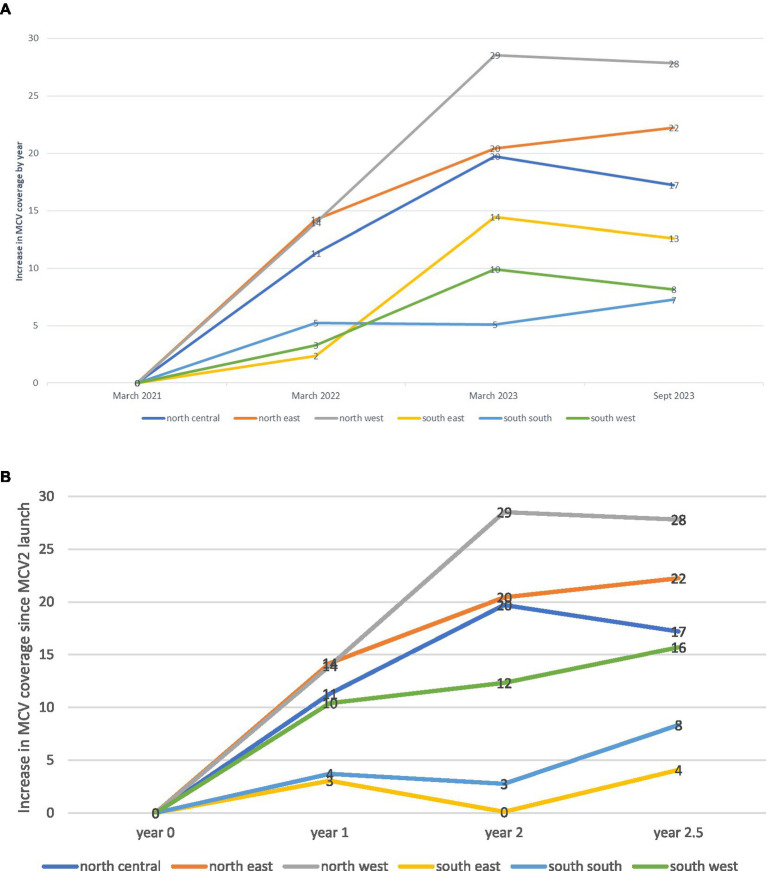
Increase in MCV2 coverage over time by geopolitical zone (%). (A) Increase in MCV2 coverage post-launch: absolute time by calendar year by geopolitical zone (percentage points). (B) Increase in MCV2 coverage post-launch: relative time from the launch date by geopolitical zone (percentage points).

Figure 4A presents the increase in MCV2 coverage over time by year and geopolitical zone. From the northern launch in March 2021 to September 2023, all zones increased their MCV2 coverage. Northern zones saw the largest increase; 28 percentage points in the northwest (*p*-value<0.001), 22 in the northeast (*p* < 0.001), and 17 in the north central (*p* = 0.013). In the south, the coverage was 13 percentage points in the southeast (*p* = 0.035), 8 in the southwest (*p* = 0.211), and 7 in the south–south zone (*p* = 0.175).

Similar to Figures 3B, 4B presents the increase in MCV2 coverage since its launch as part of the RI campaign, accounting for different launch timings by zone. The pattern aligns with Panel A, with the highest increases seen in three northern zones.

## Discussion

This study evaluated the time trend of MCV2 coverage by geopolitical zone in Nigeria, from its launch as part of the RI campaign in late 2019 to September 2023. MCV2 coverage increased across all zones between 2020 and 2023. Although initial coverage did not differ significantly by zone, northern zones observed a substantially larger increase after 2 years compared to southern zones.

Measles prevalence has been higher in northern zones, especially the northeast and northwest, over the past decade (4). Northern zones also face lower vaccination rates, with the northwest recording the lowest MCV1 coverage in the country at 39% (8). In contrast, the southeast zone, with 75% < CV1 coverage, has not seen large-scale measles outbreaks recently.

The increased MCV2 coverage in northern Nigeria is encouraging especially because the region faces the high measles burden. The greater rise in MCV2 coverage in northern zones suggests that MCV2 introduction via the RI campaign may help reduce health inequities by improving measles vaccine uptake in northern zones more than in southern zones.

The literature shows that introducing new vaccines can have mixed effects on immunization coverage (11). A review by Hyde et al. (12) found that new vaccine introductions strengthened surveillance and registry systems in countries such as the United Kingdom, Canada, and South Africa. However, most evidence comes from high-income countries, and new vaccine introductions could negatively impact coverage in low-income countries with weaker infrastructure. None of these studies, however, evaluated the effect of new vaccines on RI schedules quantitatively.

Finally, as MCV2 was recently introduced, it is crucial to monitor vaccination coverage over time to ensure no child is left behind, particularly by maintaining the focus on vulnerable populations with effective interventions such as MCV2 programs.

## Conclusion

The introduction of MCV2 as part of the RI schedule has potentially narrowed down the health inequity among the Nigerian population. Future studies should continue monitoring the progress and saturation of the MCV2 across Nigeria.

## Data Availability

The data analyzed in this study is subject to the following licenses/restrictions: application should be made to National Primary Healthcare development agency in Nigeria to access the datasets. Requests to access these datasets should be directed to https://nphcda.gov.ng/.
